# Applicability of respiratory variations in stroke volume and its surrogates for dynamic fluid responsiveness prediction in critically ill patients: a systematic review of the prevalence of required conditions

**DOI:** 10.5935/0103-507X.20170011

**Published:** 2017

**Authors:** Leandro Utino Taniguchi, Fernando Godinho Zampieri, Antonio Paulo Nassar Jr.

**Affiliations:** 1 Disciplina de Emergências Clínicas, Faculdade de Medicina, Universidade de São Paulo - São Paulo (SP), Brasil.; 2 Instituto de Ensino e Pesquisa, Hospital Sírio-Libanês - São Paulo (SP), Brasil.; 3 Unidade de Terapia Intensiva, Hospital Alemão Oswaldo Cruz - São Paulo (SP), Brasil.; 4 Unidade de Terapia Intensiva de Adultos, A.C. Camargo Cancer Center - São Paulo (SP), Brasil.

**Keywords:** Critical care, Monitoring, physiologic, Hemodynamics, Fluid therapy

## Abstract

**Objective:**

The present systematic review searched for published data on the prevalence of required conditions for proper assessment in critically ill patients.

**Methods:**

The Medline, Scopus and Web of Science databases were searched to identify studies that evaluated the prevalence of validated conditions for the fluid responsiveness assessment using respiratory variations in the stroke volume or another surrogate in adult critically ill patients. The primary outcome was the suitability of the fluid responsiveness evaluation. The secondary objectives were the type and prevalence of pre-requisites evaluated to define the suitability.

**Results:**

Five studies were included (14,804 patients). High clinical and statistical heterogeneity was observed (I^2^ = 98.6%), which prevented us from pooling the results into a meaningful summary conclusion. The most frequent limitation identified is the absence of invasive mechanical ventilation with a tidal volume ≥ 8mL/kg. The final suitability for the fluid responsiveness assessment was low (in four studies, it varied between 1.9 to 8.3%, in one study, it was 42.4%).

**Conclusion:**

Applicability of the dynamic indices of preload responsiveness requiring heart-lung interactions might be limited in daily practice.

## Abstract (pt)

**Objetivo:**

Avaliar os dados publicados em relação à prevalência das condições requeridas para avaliação apropriada em pacientes críticos.

**Métodos:**

Foram realizadas buscas nas bases de dados MEDLINE, *Scopus* e *Web of Science* para identificar estudos que discutiam a prevalência de condições validadas para avaliação da responsividade a fluidos com uso de variações respiratórias do volume sistólico ou algum outro substituto em pacientes críticos adultos. O desfecho primário foi a prevalência de adequação para avaliação da responsividade. O objetivo secundário foi o tipo e a prevalência de pré-requisitos avaliados para definir a adequação.

**Resultados:**

Incluíram-se cinco estudos (14.804 pacientes). Observaram-se elevadas heterogeneidades do ponto de vista clínico e estatístico (I^2^ = 98,6%), o que impediu o agrupamento dos resultados em uma conclusão sumarizada significativa. A limitação mais frequentemente identificada foi a ausência de ventilação mecânica invasiva com volume corrente ≥ 8mL/kg. A adequação final para avaliação da responsividade a fluidos foi baixa (em quatro estudos, variou entre 1,9 e 8,3% e, em um estudo, foi de 42,4%).

**Conclusão:**

A aplicabilidade na prática diária de índices dinâmicos de responsividade da pré-carga que demandam interações cardiopulmonares pode ser limitada.

## INTRODUCTION

Fluid resuscitation is one of the most important interventions in patients with acute circulatory failure. Volume expansion is expected to be of hemodynamic benefit if the increase in the cardiac preload is accompanied by an increase in the stroke volume to a similar extent (preload responsiveness).^(1,2)^ This improvement in the cardiac output is expected to ameliorate perfusion deficits if administered in a timely manner.^(3)^ However, positive fluid balance is increasingly associated with morbidity and mortality in critical illness.^(4-6)^ Therefore, fluid administration should be titrated by accurate parameters, such as dynamic indices of fluid responsiveness (e.g., stroke volume variation).^(7,8)^

One major constraint of most of these dynamic parameters is the requirement for invasive mechanical ventilation with the controlled mode and adequate tidal volume (Vt).^(9)^ Other requirements are sinus rhythm, the presence of an arterial line and appropriate monitoring devices.^(8)^ These limitations could undermine the bedside applicability of these parameters. In fact, some studies suggest that this might be the case.^(10,11)^ The objective of this systematic review was to estimate the prevalence of required conditions for proper use of the stroke volume variation (SVV) or other similar surrogates (e.g., pulse pressure variation [PPV]) of fluid responsiveness in critically ill patients.

## METHODS

### Literature search

Studies were identified through a standardized search of Medline (via PubMed), Scopus and Web of Science databases. A sensitive search strategy was used, which combined the following keywords: "fluid responsiveness" or "preload responsiveness" or "volume responsiveness" and "prevalence" or "incidence" or "applicability" or "suitability". The references in the included studies and personal files were also searched. The search strategy was restricted to studies that aimed to assess the fluid responsiveness in adult subjects and published prior to December 1, 2015. There was no language restriction. The titles and abstracts were assessed for eligibility, and full-text copies of all articles deemed potentially relevant were retrieved. A standardized eligibility assessment was independently performed by two reviewers. Disagreements were resolved by consensus. The PRISMA statement was used for guidance,^(12)^ and the systematic review was registered in the PROSPERO database (CRD42016032769).

### Study selection

Studies that fulfilled the following criteria were included: aimed to assess the prevalence of validated conditions for fluid responsiveness assessment using the SVV or another surrogate in a population of critical care or surgical adult patients; described the proportion of patients with the following fundamental conditions to assess the fluid responsiveness: invasive mechanical ventilation, absence of breathing efforts, sinus rhythm, "adequate Vt" (as defined by each study) and threshold used to define its adequacy.

### Data extraction

A data extraction sheet was developed. Two authors independently extracted the following data from the included studies: year of publication, country, study type (cross-sectional or cohort) and total number of assessed patients. Out of the total number of patients, the proportion of patients on invasive mechanical ventilation, without breathing efforts and with sinus rhythm was recorded. Additionally, if available, we collected data on the number of patients with an arterial line, vasopressors, cutoff for positive end expiratory pressure (PEEP) and for Vt that were considered unsuitable to assess fluid responsiveness (and the number of patients ventilated with lower levels from that cutoff), heart rate to respiratory rate ratio (HR/RR) > 3.6^(13)^ and total respiratory system compliance (C_TRS_) > 30mL/cmH_2_O.^(14)^

The risk of bias in the individual trials was not assessed because we only planned on including prevalence studies and commonly evaluated variables in the quality assessment, such as selection of cases, controls or cohorts, ascertainment of exposures and follow-up of the patients were not assessed in these prevalence studies.

### Outcome measurement

The primary outcome was the prevalence of suitability for assessing the fluid responsiveness, defined as the number of patients who were invasively, mechanically ventilated with a Vt higher than the identified threshold, who lacked breathing efforts and had a regular sinus rhythm.

A formal meta-analysis was planned, but it was not performed because of the heterogeneity among the studies (I^2^ = 98.6%).^(15)^

## RESULTS

**Figure 1:**
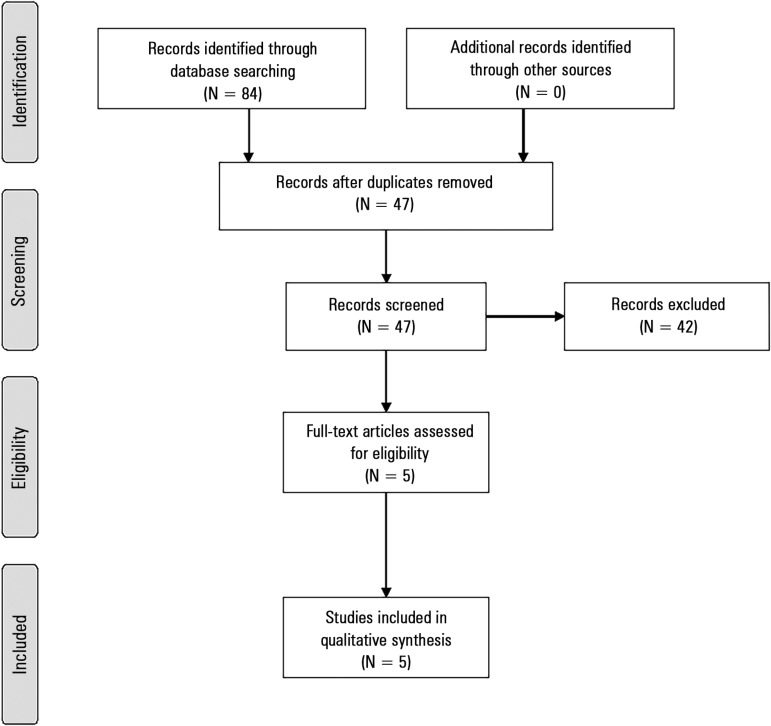
Study flowchart.

Of 84 publications retrieved through electronic database searches, five studies were included (Figure 1).^(10,11,16-18)^ There were one prospective,^(11)^ two retrospective^(16,17)^ and two one one-day point prevalence studies.^(10,18)^ One study was performed in a surgical room^(16)^ and the remaining were all performed in intensive care units (ICU). The study by Benes et al. selected a population in which required conditions for preload responsiveness were only assessed in patients with the following conditions: sepsis, trauma, postoperative and post-cardiac arrest.^(17)^ Three studies included patients from more than one center (Table 1).

**Table 1:** Characteristics of the included studies

Study	Country	Type of study	Setting	Number of centers
Mahjoub et al.^(10)^	France	Cross-sectional	ICU	26
Mendes et al.^(11)^	Brazil	Prospective	ICU	2
Maguire et al.^(16)^	USA	Retrospective	Surgical room	1
Benes et al.^(17)^	Czech Republic	Retrospective	ICU	1
Fischer et al.^(18)^	France	Cross-sectional	ICU	36

ICU - intensive care unit.

The characteristics of the assessed patients in included studies are given in table 2. A total of 14,804 patients were evaluated. Overall, except for one study, more than half of patients were mechanically ventilated. However, only one study reported that the majority of patients lacked a breathing effort. We also observed that the use of arterial lines varied among the studies (15.7 to 81%) as did the administration of vasopressors. Their use varied from only 192 of 4,792 patients (4%), which had the required conditions for assessing fluid responsiveness in the study by Maguire et al.^(16)^ to 59% of critically ill patients in the study by Benes et al.^(17)^ All other studies of critical care patients had a lower use of vasopressors (13.5 to 28.5%).

**Table 2:** Prevalence of conditions affecting the assessment of fluid responsiveness in the included studies

Study	Number of patients	Mechanical ventilation	Controlled mechanical ventilation	Tidal volume ≥ 8mL/kg	Sinus rhythm	Arterial line	Suitability for assessment of fluid-responsiveness
Mahjoub et al.^(10)^	311	158 (50.8)	44 (14.1)	12 (3.8)	274 (88.1)	170 (54.7)	12 (3.8)
Mendes et al.^(11)^	424	106 (25.0)	33 (7.8)	12 (2.8)	404 (95.2)	69 (16.3)	12 (2.8)
Maguire et al.^(16)^	12,308	7,754 (63.0)	NA	5,046 (41.0)	NA	1,936 (15.7)	4,792 (38.9)† 1,019 (8.3)‡
Benes et al.^(17)^	1,296	1,073 (82.8)	983 (75.8)	585 (45.1)	1,191 (91.9)	1,050 (81.0)	549 (42.4)
Fischer et al.^(18)^	465	282 (60.6)	127 (27.3)	25 (5.4)	408 (87.7)	324 (69.7)	9 (1.9)

NA - not available.

† data for respiratory variations in the plethysmographic waveform amplitude.

‡ data for the pulse pressure variation. Data presented as the number of patients (percentage of the total number of patients).

Two studies reported a PEEP value cutoff for assessing the fluid responsiveness. Maguire et al.^(16)^ defined it as 5cmH_2_O, and 56.4% of all mechanically ventilated patients had a PEEP equal to or lower than that. Benes et al.^(17)^ defined the PEEP cutoff as 10cmH_2_O, and 52.9% of mechanically ventilated patients had a PEEP level equal to or lower than that. All identified studies used a threshold of 8mL/kg as the cutoff of validity for the SVV or surrogate.

Mahjoub et al.^(10)^ also gathered data on other physiological criteria for assessing the fluid responsiveness and found that 10 (3.2%) patients had an HR/RR > 3.6, and 8 (2.6%) patients had a C_TRS_ > 30mL/cmH_2_O. Fischer et al.^(18)^ found that 177 (38.1%) patients an HR/RR > 3.6 and 108 (23.2%) patients had a C_TRS_ > 30mL/cmH_2_O. Additionally, they considered a tricuspid annular peak systolic velocity > 0.15ms^-1^ as suitable for the preload responsiveness assessment and only six (2%) patients fulfilled these criteria as well the other required conditions for this assessment (mechanical ventilation, regular rhythm, no spontaneous breathing, Vt > 8mL/kg, HR/RR > 3.6 and C_TRS_ > 30mL/cmH_2_O).

Overall, the prevalence of the required conditions, i.e., invasive mechanical ventilation, absence of breathing efforts, Vt higher than the identified threshold (8mL/kg of body weight in all studies) and sinus rhythm, was very low in three ICU studies (1.9 to 3.8%). In contrast, two studies found a higher proportion (38.9 and 42.4%) of patients presenting with the required conditions for assessing the fluid responsiveness. One of these studies only included surgical patients^(16)^ and the other included a selected population of critical care patients, as mentioned above (Table 2).^(17)^ Of note, the study by Maguire et al.^(16)^ assessed the proportion of patients fulfilling criteria for both respiratory variations in the plethysmographic waveform amplitude (38.9% from the total population) and PPV (8.3% from the total population).

## DISCUSSION

Since "dynamic" parameters (such as SVV and PPV) have been advocated to have greater accuracy in predicting the fluid responsiveness,^(1,19,20)^ their bedside applicability in the real world context has become a relevant question due to their known constrains. In this systematic review, we could observe the following: (1) there is a paucity of studies about the prevalence of requisites for correct application of respiratory-dependent dynamic parameters; (2) the available literature has a marked heterogeneity; and (3) at most, these parameters could be applied to 42% of the patients in the ICU, which is usually to less than 10%.

After the Michard et al. publication on the utility of PPV in the early 2000s,^(21)^ substantial enthusiasm was observed about dynamic indices to predict fluid responsiveness. However, many limitations for the use of respiratory variations in stroke volume or surrogates have been identified. The most relevant one is the absolute requirement for the absence of spontaneous respiratory efforts (i.e., invasive mechanical ventilation in the controlled mode).^(22,23)^ We observed high variability in the prevalence of invasive mechanical ventilation (from 25 to 82.8%), which is probably due to the case-mix among studies. Of note, the study from Benes et al., which demonstrated the highest proportion of mechanically ventilated patients, only evaluated a highly selected severe subgroup, as previously discussed.^(17)^ Even the study by Mendes et al.,^(11)^ which had the lowest prevalence of invasive mechanical ventilation (25%), presented values that were similar to a large multicenter cohort study of mechanical ventilation.^(24)^ More recently, the LUNG-SAFE study evaluated 459 ICU in 50 different countries and observed 46.5% of critically ill patients underwent invasive mechanical ventilation.^(25)^

In addition to the absence of respiratory efforts, another limitation is the requirement for a certain level of variation in the intrathoracic positive pressure due to the tidal volume (usually a threshold of Vt ≥ 8mL/kg, as observed in our systematic review).^(9,26)^ We observed that the proportion of critically ill patients with invasive mechanical ventilation and tidal volumes higher than 8mL/kg is low (usually less than 10% in three of the included studies). This might be due to the recent literature, which demonstrated that even small periods in susceptible patients of non-protective ventilation could induce harm.^(27-29)^ Other constraints are the absence of a cardiac arrhythmia and presence of an arterial line, whose insertion practice is also highly variable between units, with median usage rates in American ICUs as low as 22.4% in medical units and 51.7% in patients with vasopressors.^(30)^ Therefore, the prevalence of required conditions for the correct application of respiratory dependent indices of fluid responsiveness is very low; commonly, it was less than 10% in the included studies (Table 2). If other confounders are also evaluated (such as HR/RR > 3.6,^(13)^ low respiratory compliance,^(14)^ intra-abdominal hypertension,^(31)^ and pulmonary hypertension^(32)^), much lower values are expected, limiting the bedside applicability of these hemodynamic evaluations.

One may argue that a formal meta-analysis to summarize the results should have been attempted. However, given the high statistical heterogeneity detected, any attempt to pool the results could be misleading. A relevant clinical heterogeneity between selected studies could be observed with the case-mix of medical and surgical patients, local setting (ICU or surgical room), different definitions of suitability for final application of dynamic parameters, and length of stay at the time of evaluation. This should be acknowledged when interpreting the final percentage of patients with valid conditions for SVV or PPV, ranging from 1.9% to 42.4%. The Cochrane Group suggests that if significant heterogeneity is detected, one possibility is to not pool the data.^(15)^ Unfortunately, due to the limited number of studies, meta-regression, which is another option, might also be misleading.

Others may also argue that in the early phases of fluid resuscitation, when volume administration has the largest microcirculatory effects,^(3)^ the presence of required conditions would probably be more frequent in the most critically ill. In fact, the only study identified in our systematic review to specifically address this early phase is also the one with the highest prevalence (the study from Benes et al.^(17)^ in table 2). However, even in septic patients, for whom timely administration of fluids is considered one of the most life-saving interventions,^(2)^ three recent large randomized controlled trials regarding protocolized early hemodynamic care observed that approximately 20% of included patients had invasive mechanical ventilation in the first 6 hours.^(33-35)^ As a result, even in this important early phase, only a minority would be correctly evaluated using dynamic parameters. Some alternatives have been published for application in patients with spontaneous breathing activity regardless of the cardiac rhythm. The passive leg raise is a preload-modifying maneuver that has been demonstrated to be an excellent predictor of fluid responsiveness (pooled area under the receiver-operating characteristics curve of 0.95 in a recent meta-analysis).^(36)^

One final remark is the observation in some recent literature that it might be accurate to apply PPV even in acute respiratory distress syndrome patients who are ventilated with low Vt.^(37,38)^ In such a population, higher PPV values might be predictive of fluid responsiveness, which is probably due to lower intrathoracic pressure variation induced by low Vt mechanical ventilation. Therefore, even with "protective ventilation", PPV (and probably SVV) could be justified with the application of higher thresholds. Nevertheless, some drawbacks should also be highlighted. Biais et al. applied the "gray zone" approach to a large cohort of mechanically ventilated patients and observed that in 62% of them, values between 4 and 17% could not predict fluid responsiveness.^(39)^ Even if one applies this rationale to weigh the benefit/risk ratio of giving/withholding fluid infusion (i.e., decide to infuse fluids in patients with high values of PPV to correct underperfusion even if they are receiving protective ventilation), fluid administration has a time-dependent effect on the microcirculation.^(3)^ Therefore, in the early phases of fluid management, application of PPV to titrate fluid infusion (such as in the operative room) may improve outcomes,^(40)^ but it could later lead to fluid accumulation without perfusion improvement.^(3)^

Our study has some strengths and limitations. First, we performed an extensive and systematic literature search for possible articles. Unfortunately, only five studies could be included and, due to the heterogeneity, a formal pooled analysis could not be performed. Second, the population studied in the included articles was treated at the surgical room and ICU, which increases the generalizability as well as the heterogeneity. Finally, to the best of our knowledge, this is the first systematic review on the prevalence of respiratory-dependent dynamic indices of fluid responsiveness. Nevertheless, even this important theme was studied in just a few articles, which highlights a relevant lack of knowledge of this issue in different settings.

## CONCLUSION

The applicability of dynamic indices of preload responsiveness that require heart-lung interactions might have limited clinical utility. More data are required on how to properly guide volume resuscitation in critically ill patients.
